# Comparison of the Efficacy of ECMO With or Without IABP in Patients With Cardiogenic Shock: A Meta-Analysis

**DOI:** 10.3389/fcvm.2022.917610

**Published:** 2022-07-07

**Authors:** Ping Zeng, Chaojun Yang, Jing Chen, Zhixing Fan, Wanyin Cai, Yifan Huang, Zujin Xiang, Jun Yang, Jing Zhang, Jian Yang

**Affiliations:** ^1^Department of Cardiology, The First College of Clinical Medical Science, China Three Gorges University and Yichang Central People's Hospital, Yichang, China; ^2^Hubei Key Laboratory of Cardiology, Department of Cardiology, Cardiovascular Research Institute, Renmin Hospital, Wuhan University, Wuhan, China

**Keywords:** extracorporeal membrane oxygenation (ECMO), intra-aortic balloon pump (IABP), cardiogenic shock, survival (MeSH), meta-analysis

## Abstract

**Objective:**

Studies on extracorporeal membrane oxygenation (ECMO) with and without an intra-aortic balloon pump (IABP) for cardiogenic shock (CS) have been published, but there have been no meta-analyses that compare the efficacy of these two cardiac support methods. This meta-analysis evaluated the outcomes of these two different treatment measures.

**Methods:**

The PubMed, Embase, Cochrane Library, Web of Science, and Clinical Trials databases were searched until March 2022. Studies that were related to ECMO with or without IABP in patients with CS were screened. Quality assessments were evaluated with the methodological index for nonrandomized studies (MINORS). The primary outcome was in-hospital survival, while the secondary outcomes included duration of ECMO, duration of ICU stay, infection/sepsis, and bleeding. Revman 5.3 and STATA software were used for this meta-analysis.

**Results:**

In total, nine manuscripts with 2,573 patients were included in the systematic review. CS patients who received ECMO in combination with IABP had significantly improved in-hospital survival compared with ECMO alone (OR = 1.58, 95% CI = 1.26–1.98, *P* < 0.0001). However, there were no significant differences in the duration of ECMO (MD = 0.36, 95% CI = −0.12–0.84, *P* = 0.14), duration of ICU stay (MD = −1.95, 95% CI = −4.05–0.15, *P* = 0.07), incidence of infection/sepsis (OR = 1.0, 95% CI = 0.58–1.72, *P* = 1.0), or bleeding (OR = 1.28, 95% CI = 0.48–3.45, *P* = 0.62) between the two groups of patients with CS.

**Conclusion:**

ECMO combined with IABP can improve in-hospital survival more effectively than ECMO alone in patients with CS.

## Introduction

Low cardiac output and hypoperfusion are highly associated with multiorgan damage and are typical manifestations of cardiogenic shock (CS). Moreover, treating CS is challenging for the attending doctors in the intensive care units (ICUs) (1). Cardiogenic shock is a life-threatening condition and patients with CS tend to have high rates of mortality (2). Mechanical heart assist devices are often needed to save patients' lives because drug therapy may be ineffective. To maintain the circulation state of CS patients and ensure the perfusion of important organs, extracorporeal membrane oxygenation (ECMO) and intra-aortic balloon pumps (IABPs) are frequently used as rescue options.

ECMO has been frequently used to treat CS in patients with myocardial infarction, explosive myocarditis, and sudden cardiac arrest (3–5). During the procedure, the venous blood is drawn out to achieve full oxygenation and then injected into the artery at a certain flow rate to ensure that the patient has an adequate oxygen supply and mean arterial pressure. An intra-aortic balloon pump (IABP) could increase diastolic pressure, augment coronary perfusion, and decrease systolic afterload, in addition to increasing the forward flow by inflating and deflating the balloon which decreases myocardial oxygen demand and provides hemodynamic support (6). ECMO and IABP can play positive roles in the treatment of CS. Intervention studies on ECMO with or without IABP are needed to improve the clinical treatment and survival rates of CS patients. The combination of these two methods has been gradually applied when rescuing patients with CS, and studies on patient survival or death have been published. However, there was no meta-analysis to evaluate the efficacy of ECMO combined with IABP and ECMO alone. In this study, we intend to systematically compare the outcomes of ECMO combined with IABP and ECMO alone in the treatment of patients with CS.

## Data and Methods

### Literature Search Strategy

PubMed, Embase, Cochrane Library, Web of Science, and Clinical Trials databases were used in the retrieval process. A combination of ECMO, intra-aortic balloon pump, and cardiogenic shock were the medical subject headings (MeSH) keywords that were searched in the English language articles. All literature in the database was searched until March 2022. The search strategy for PubMed and other databases is described in the Supplementary Material. This meta-analysis was conducted according to the PRISMA recommendation (Preferred Reporting Items for Systematic Reviews and Meta-Analysis) (7).

### Literature Inclusion and Exclusion Criteria

Literature inclusion criteria: (1) patients diagnosed with CS; (2) patients treated with ECMO alone vs. ECMO plus IABP; and (3) intervention studies. Exclusion criteria: (1) meta-analyses, reviews, comments, and letters; (2) literature without basic data; (3) literature with duplicate data; and (4) articles on animal experiments. This systematic review was performed by two authors who have previously published multiple meta-analyses. Each author independently judged whether the retrieved literature could be included in the study, and an additional judgment by a third author was provided in case of disagreement.

### Literature Quality Evaluation Criteria

The methodological index for nonrandomized studies (MINORS) was applied for an in-depth assessment of the quality of the research (8). The index was used to evaluate the quality of the literature by assigning values to 12 items, each of which was scored 0 (not reported), 1 (reported but inadequate), or 2 (reported and adequate). The total MINORS scores were obtained by adding all the scores of the 12 items. Moreover, two authors independently evaluated the MINORS scores for each included study (Table 1).

**Table 1:** Characteristics of the included studies.

**References**	**Country**	**Centers**	**Methods**	**Age**	**Male**	**BMI**	**Diabetes**	**Hypertension**	**COPD**	**MI**	**PCI**	**CABG**	**Myocarditis**	**Lactic acid**	**MINORS**
		**(*n*)**			***n*** **(%)**		***n*** **(%)**	***n*** **(%)**	***n*** **(%)**	***n*** **(%)**	***n*** **(%)**	***n*** **(%)**	***n*** **(%)**	***n*** **(%)**	
van den Brink et al. (9)	Europe	Multicenter	ECMO	59 ± 7	9 (82)	29.1 ± 3.8	1 (9)	5 (45)	.	.	0 (0)	.	.	.	18
			ECMO+IABP	59 ± 11	8 (86)	24.5 ± 1.98	3 (43)	1 (14)	.	.	2 (29)	.	.	.	17
Djordjevic et al. (10)	Germany	Single	ECMO	64 (55, 73)	24 (56)	26 (23, 32)	14 (33)	.	6 (14)	.	.	15 (35)	.	7.4 (6.2, 14.9)	19
			ECMO+IABP	66 (55, 73)	102 (79)	27 (25, 30)	40 (31)	.	13 (10)	.	.	100 (78)	.	9.0 (4.9, 14.1)	18
Bjornsdottir et al. (11)	Sweden	Multicenter	ECMO	63 ± 13	328 (66)	27 ± 5	108 (22)	.	.	146 (29)	72 (15)	214 (43)	.	.	16
			ECMO+IABP	62 ± 15	82 (71)	27 ± 5	33 (28)	.	.	37 (32)	22 (19)	57 (49)	.	.	18
Brechot et al. (12)	France	Single	ECMO	49 (34, 57)	100 ± 64.5	25 (22, 29)	.	.	.	60 (38.7)	.	.	26 (16.8)	9.5 (6.0, 13.7)	16
			ECMO+IABP	52 (44, 61)	81 ± 77.9	26 (23, 29)	.	.	.	68 (65.4)	.	.	6 (5.8)	7.0 (4.6, 10.6)	19
Barge-Caballero et al. (13)	Spain	Multicenter	ECMO	50.9 ± 13.3	73 (76)	26 ± 4.2	26 (27.1)	.	4 (4.2)	21 (21.9)	.	.	.	.	18
			ECMO+IABP	49.4 ± 12.7	55 (75.3)	25.3 ± 5.1	17 (23.3)	.	3 (4.1)	33 (45.2)	.	.	.	.	16
Kida et al. (14)	Japan	Multicenter	ECMO	70.84 ± 11.01	39 (66.1)	22.86 (3.3)	13 (27.1)	.	.	7 (14.3)	43 (81.1)	2 (4.1)	.	.	19
			ECMO+IABP	66.35 (12)	367 (80.1)	24.09 (3.82)	173 (42.7)	.	.	64 (14.8)	405 (93.1)	23 (5.9)	.	.	18
Char et al. (15)	New York	Single	ECMO	58 (48, 70)	83 (58)	.	43 (30.1)	95 (66.4)	6 (8.8)	24 (16.8)	.	.	1 (0.7)	4.5 (1.7, 9.9)	16
			ECMO+IABP	59.5 (47, 68.5)	47 (69.1)	.	19 (27.9)	39 (57.4)	15 (10.5)	15 (22.1)	.	.	6 (8.8)	3.2 (1.8, 5.6)	16
Lin et al. (16)	Taiwan	Single	ECMO	52.8 ± 17.2	158 (70)	23.9 ± 4.3	60 (26.4)	66 (29.1)	4 (1.8)	19 (8.4)	.	30 (13.2)	35 (15.4)	3.0 (2, 4.4)	18
			ECMO+IABP	56.8 ± 13.4	240 (79.5)	25.1 ± 3.9	111 (36.8)	119 (39.4)	3 (1)	28 (9.3)	.	116 (38.4)	33 (10.9)	3.1 (2.1, 4.6)	18
Monaco et al. (17)	Italy	Single	ECMO	67 (60, 73)	69 (90.7)	25.83 ± 4.58	12 (15.8)	42 (55.3)	.	.	.	8 (10.5)	.	1.33 (1.12, 1.91)	18
			ECMO+IABP	66 (59, 71)	43 (95.6)	26.23 ± 5.29	11 (24.4)	24 (53.3)	.	.	.	5 (11.1)	.	1.28 (0.91, 1.67)	16

*BMI, body mass index; COPD, chronic obstructive pulmonary disease; PCI, percutaneous coronary intervention; MI, Myocardial infarction; CABG, coronary artery bypass grafting; ECMO, extracorporeal membrane oxygenation; IABP, intra-aortic balloon pump*.

### Data Extraction

We extracted the baseline information of the studies, including authors, publication year, country, age, sex composition, body mass index (BMI), patients with diabetes, hypertension, chronic obstructive pulmonary disease (COPD), prior PCI, prior myocardial infarction (MI), a priori coronary artery grafting (CABG), and several other items. Data that was not directly extracted was obtained by data transformation.

### Statistical Methods

Statistical analysis of the data was performed using RevMan 5.3 software. We used the fixed or random-effects meta-analysis model and a forest plot to present the pooled estimates of the odds ratio (OR) or mean difference (MD) and 95% confidence interval (CI). The heterogeneity was evaluated using the *I*-square (*I*^2^) statistic. A funnel map was established to assess publication bias, and STATA 13.0 software was used for sensitivity analysis. *P-*value < 0.05 was considered a statistically significant difference.

## Results

### Literature Search Results

**Figure 1:**
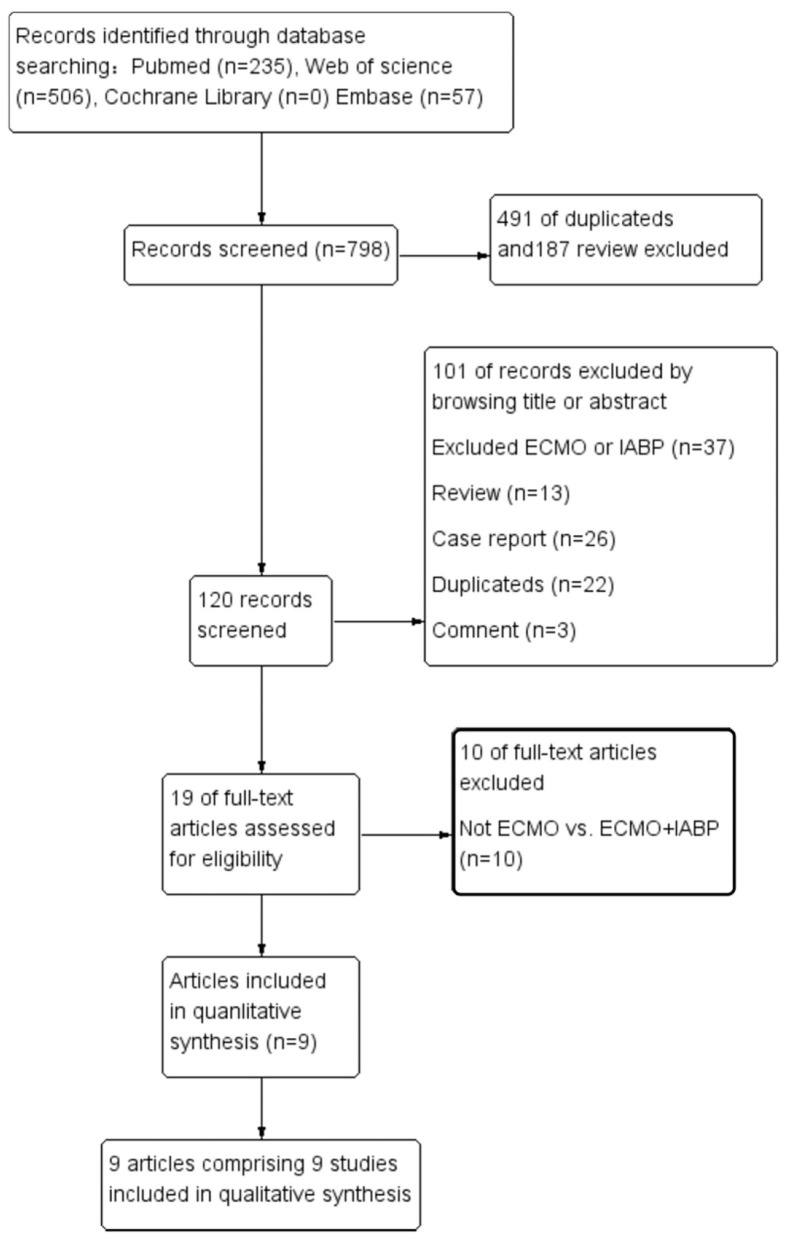
Flowchart of the study selection process in the meta-analyzes.

Seven hundred and ninety-eight articles were obtained by searching with the proposed criteria. A total of 491 duplicate articles and 187 review-type articles were then excluded by reading the titles and abstracts. A total of 120 articles were included in the next retrieval step, and 101 articles were eliminated after carefully reading the full texts. Ultimately, a total of 19 articles were carefully investigated, and 10 of them were excluded because they were not intervention studies (Figure 1). The flowchart of the study selection process was conducted according to the PRISMA recommendation (7).

### Basic Characteristics of the Included Literature

A total of nine studies (9–17) were ultimately included, including studies from New York, Japan, Taiwan, and cities in Europe, with a total of 2,573 patients (Table 1). The basic characteristics of the participants were as follows: the proportion of men was between 66 and 95; the mean BMI (body mass index) ranged from 22.8 to 29.1 kg/m^2^; 9–43% had diabetes; 4–66% had hypertension; 1–14% had COPD (chronic obstructive pulmonary disease), and 8–65% had MI (myocardial infarction). The indications for ECMO/IABP included myocarditis, dilated heart disease, acute heart failure, and so on (Table 1).

### Quality of Evidence and Risk of Bias Across Studies

**Figure 2:**
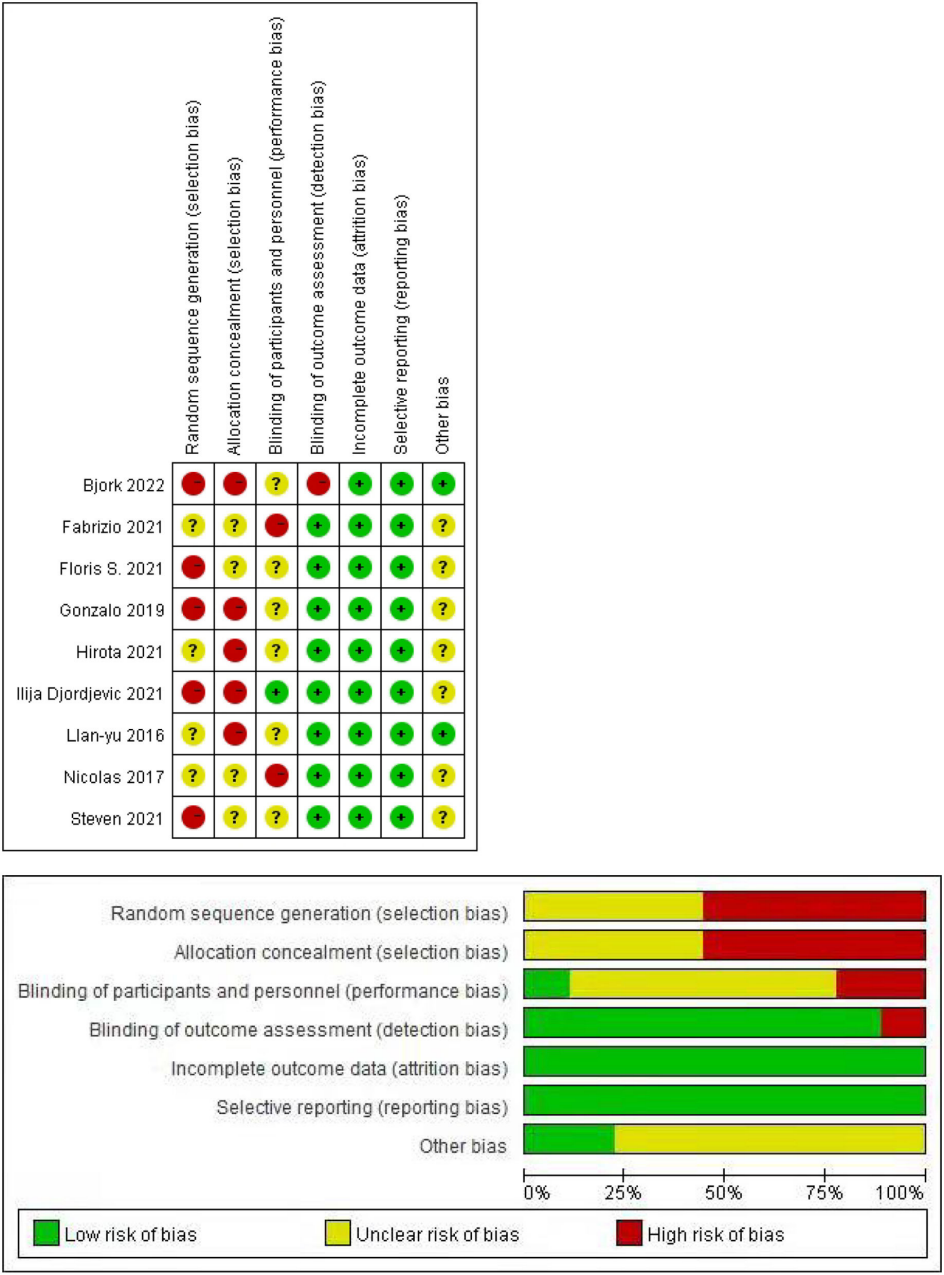
Publication bias of this meta-analysis.

The quality of evidence according to the MINORS is presented in the last line of Table 1. The total score of each article was more than 16, which indicated that the quality of the articles was relatively high. In addition, all the included studies may have publication biases. We did not include RCTs, so there are many red dots and long red bars in Figure 2, which represents a high risk of random sequence generation and allocation concealment.

### Outcomes

#### In-hospital Survival

**Figure 3:**
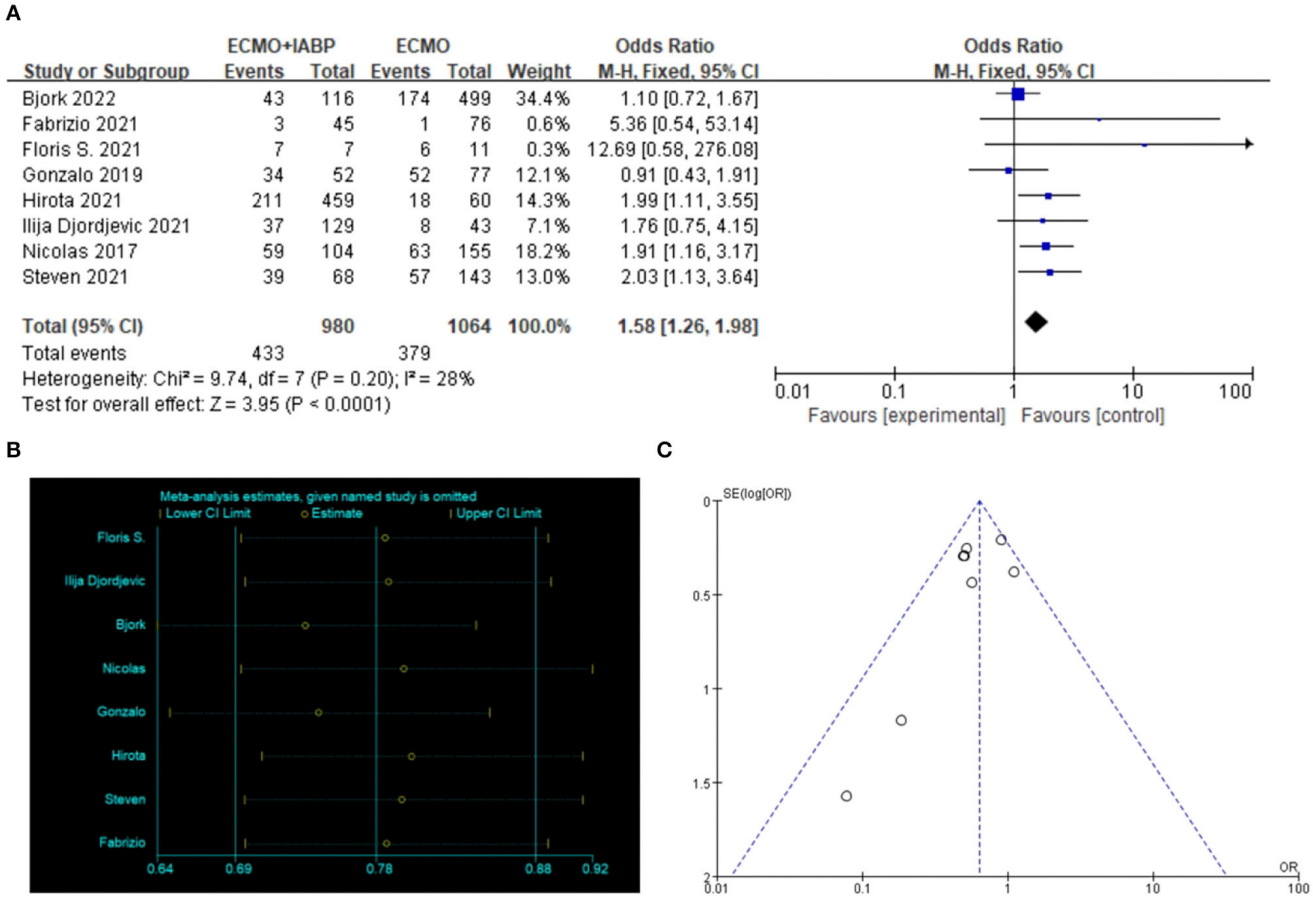
In-hospital survival. Forest plot of in-hospital survival in patients treated with ECMO with IABP vs. ECMO alone **(A)**, the sensitivity analysis was tested in STATA **(B)**. The symmetry of the funnel diagram indicates that there is no publication bias **(C)**. ECMO, extracorporeal membrane oxygenation; IABP, intra-aortic balloon pump.

ECMO combined with IABP treatment was associated with a higher rate of in-hospital survival in patients with CS (OR = 1.58, 95% CI = 1.26–1.98, *P* < 0.0001; Figure 3A). STATA software was used to verify the sensitivity analysis, and the result was stable after a single exclusion of RCTs (Figure 3B). The funnel map was stacked, and all points were under the funnel, indicating that there was no publication bias in the included studies when evaluating the result of in-hospital survival (Figure 3C).

#### Duration of ECMO and ICU Admission

**Figure 4:**
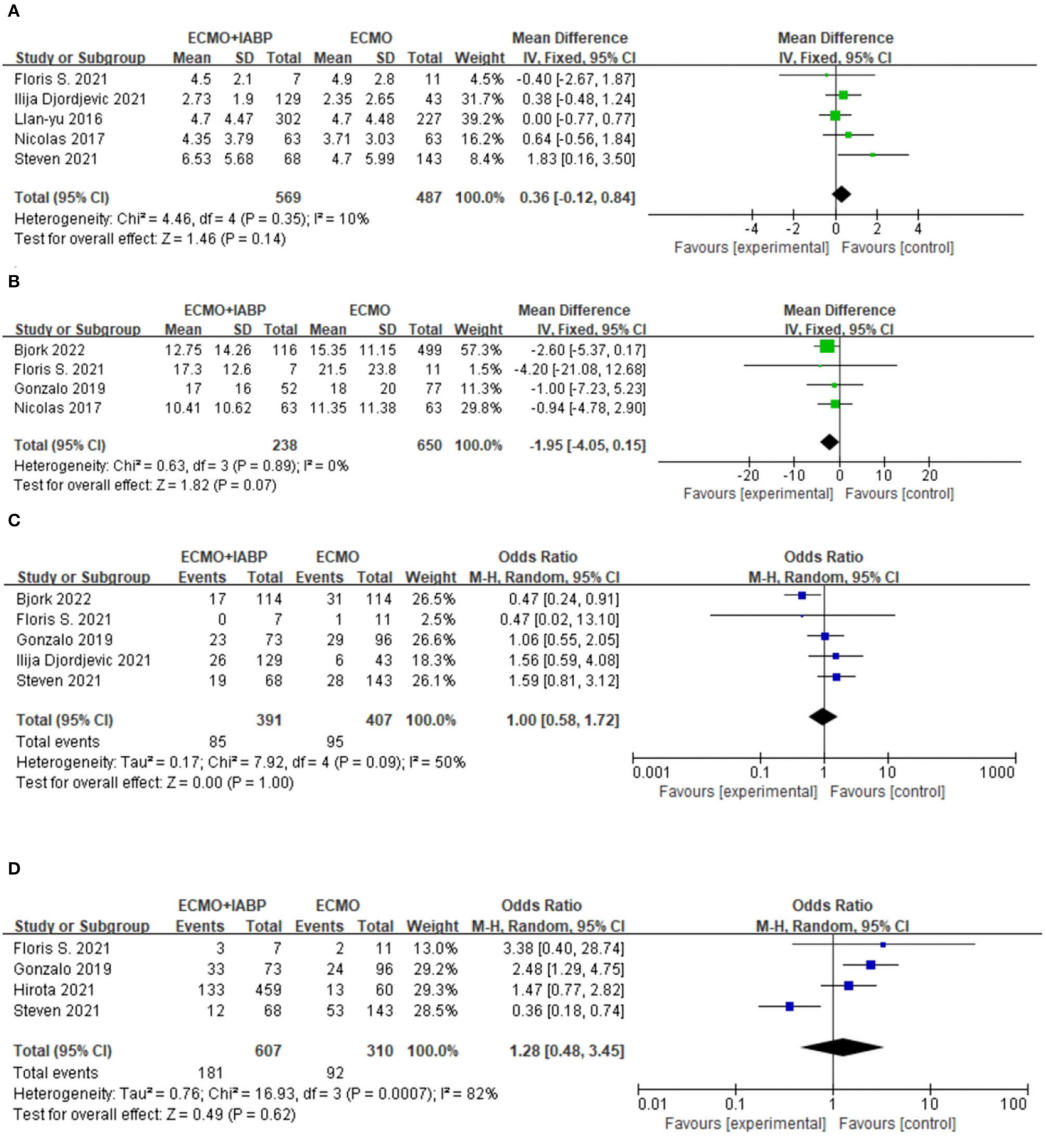
Forest plots of this meta-analysis. Duration of ECMO **(A)**, Duration of ICU **(B)**, Infection/sepsis **(C)**, and Bleeding **(D)**. ECMO, extracorporeal membrane oxygenation; IABP, intra-aortic balloon pump.

The treatment of ECMO combined with IABP did not significantly reduce the duration of ECMO (MD = 0.36, 95% CI = −0.12–0.84, *P* = 0.14; Figure 4A) or ICU stay (MD = −1.95, 95% CI = −4.05–0.15, *P* = 0.07) in the patients with CS (Figure 4B).

#### Incidence of Infection/Sepsis

There was no difference in the incidence of infection or sepsis between the two groups (OR = 1.0, 95% CI = 0.58–1.72, *P* = 1.0; Figure 4C).

#### Bleeding

There was no significant difference in the bleeding results between the two intervention methods (OR = 1.28, 95% CI = 0.48–3.45, *P* = 0.62; Figure 4D).

## Discussion

We conducted a systematic meta-analysis of the studies on ECMO with IABP vs. ECMO alone in patients with CS. After conducting the meta-analysis, we found that ECMO combined with IABP was beneficial in saving lives. We found that there was a significant increase in the in-hospital survival rate in CS patients who received ECMO combined with IABP when compared with those who received ECMO alone. There was no obvious discrepancy in the duration of ECMO and ICU stay, the incidence of infection/sepsis, or bleeding between the two groups. This meta-analysis was the first to analyze intervention studies on ECMO with IABP and ECMO alone in CS patients. The results revealed that a combined therapy of ECMO and IABP was critical to improving the in-hospital survival rate. This meta-analysis provided a reference for the clinical application of cardiac auxiliary devices.

In-hospital survival is a direct index used to evaluate the therapeutic effect of rescue measures for severe cardio-respiratory failure. IABP acted as an assisting circulatory support device that could enhance cardiac diastolic function and improve coronary (18), cerebral (19), and systemic circulation. Regardless of the cause of shock, ECMO and IABP are guaranteed to save lives. Madershahian et al. proved that additional IABP therapy was shown to increase coronary bypass graft flow (ECMO alone 46.869.6 ml/min vs. ECMO+IABP 56.4612.1 ml/min; *p* < 0.005) (20). A 10-year clinical study found that IABP alone could significantly improve the survival rate of CS patients when compared with ECMO alone (IABP = 49.5%, ECMO = 30.5%) (21). Multiple studies have proven that IABP plays an important role in improving the survival rate of patients. In our meta-analysis, we also found that IABP, as an additional therapy, can significantly improve the in-hospital survival rate in CS patients.

The duration of ECMO and ICU stay were related to the prognosis of CS patients. Omar et al. proved that the duration of ECMO was an independent predictor of intracranial hemorrhage during ECMO support. They believed that the longer the ECMO duration, the higher the probability of intracranial hemorrhage (22). Data from a large multicenter database suggested that a longer duration of ECMO support after pediatric cardiac surgery was associated with poor outcomes (23). Glenn et al. (24) found an improved survival rate but no decrease in ECMO duration (25). In our meta-analysis, we also found no shortening of ECMO or ICU duration between these two groups, with low heterogeneity among the included studies. We believe that for patient survival, the shorter the duration of ECMO and ICU stay, the better the treatment effect.

Infection or sepsis may lead to fever and increase the burden on the circulatory system. Allou et al. found that 17.7% of patients developed cannula-related infections, including Enterobacteriaceae (38%), and Staphylococcus spp. (28.2%), and Pseudomonas aeruginosa (18.3%) (25). Yun et al. found that patients with gram-negative rods died more frequently and earlier than those with gram-positive cocci (26). However, there was also evidence that proved that an acquired infection was not independently associated with mortality (27) and that a prolonged duration of ECMO was an independent risk factor for nosocomial infection (28). In our meta-analysis, we found that there was no significant difference in infection or sepsis when patients were treated with ECMO and IABP or ECMO alone. This may be related to the aseptic operation and the care of the pipeline area during and after catheterization rather than the treatment difference.

Bleeding and thrombotic complications during ECMO have a significant impact on patient outcomes (29). Optimal anticoagulation, such as antithrombin supplementation, was needed while the cardiac assist device was used after analyzing activated partial thromboplastin time (APTT), antithrombin (AT) activity, platelet count, and fibrinogen concentration (30). In our meta-analysis, there was no significant difference in bleeding between ECMO combined with IABP and ECMO alone. Bleeding might be related to the use of anticoagulants and not independently affected by the treatment measures. However, different modes of ECMO and primary diagnoses were proven to be related to total bleeding (31). There was no adequate information on thrombosis to conduct the meta-analysis in our study. Moreover, it should be noted that removal of ECMO was associated with thrombosis (31). It is important to remove the ECMO cannula first and then the IABP cannula second to prevent the showering of thrombi.

Potential limitations existed in the process of this meta-analysis. First, all the patients had pre-existing conditions, with different diseases and different levels of lactic acid, which may have affected the outcomes of the study. Moreover, there was no particular order in which the IABP and ECMO were placed, which resulted in heterogeneity. Second, the nine articles were authored from different continents like America, Europe, and Asia, which may have affected the outcomes for the CS patients. Different race populations may have different responses to the same treatment. Third, all patients had different causes of CS and various degrees of myocardial injury and elevated blood lactic acid that occurred before treatment with ECMO or IABP. Fourth, the definition of bleeding may vary in different trials. We did not perform a subgroup analysis of bleeding because of insufficient data. Fifth, the nine studies were not randomized controlled trials but were same-period nonrandom control trials, which may have had an impact on the outcomes. Although there were no RCTs in the clinical intervention study of the combination of ECMO and IABP, we believe that it was reasonable to choose additional IABP therapy according to the patient's clinical needs rather than random selection.

## Conclusion

This study showed that ECMO combined with IABP could be more effective in improving survival in patients with CS than ECMO alone.

## Data Availability Statement

The original contributions presented in the study are included in the article/Supplementary Material, further inquiries can be directed to the corresponding authors.

## Author Contributions

This study was designed by JZ and PZ. PZ and CY contributed data to the paper and statistical analysis. Interpretation of data were performed by PZ. All authors were involved in the drafting and revision of the manuscript and the final version to be published.

## Funding

This work was supported by the National Natural Science Foundation of China (82070372 and 82170418) and Hubei Province's Outstanding Medical Academic Leader Program.

## Conflict of Interest

The authors declare that the research was conducted in the absence of any commercial or financial relationships that could be construed as a potential conflict of interest.

## Publisher's Note

All claims expressed in this article are solely those of the authors and do not necessarily represent those of their affiliated organizations, or those of the publisher, the editors and the reviewers. Any product that may be evaluated in this article, or claim that may be made by its manufacturer, is not guaranteed or endorsed by the publisher.
