# Supplementary material for: Comparison of the Efficacy of ECMO With or Without IABP in Patients With Cardiogenic Shock: A Meta-Analysis
Source: Front Cardiovasc Med. 2022 Jul 7;9:917610. doi: 10.3389/fcvm.2022.917610 (PMC9300857; doi:10.3389/fcvm.2022.917610)

**PubMed（n=235）**

#1 **ECMO [MeSH Terms]**

#2 **((((((((((((((((((((Extracorporeal Membrane Oxygenat.**

**ions[Title/Abstract]) OR (Membrane Oxygenation, Extracorporeal[Title/Abstract])) OR (Oxygenation, Extracorporeal Membrane[Title/Abstract])) OR (ECMO Treatment[Title/Abstract])) OR (ECMO Treatments[Title/Abstract])) OR (Treatment, ECMO[Title/Abstract])) OR (ECLS Treatment[Title/Abstract])) OR (ECLS Treatments[Title/Abstract])) OR (Treatment, ECLS[Title/Abstract])) OR (ECMO Extracorporeal Membrane Oxygenation[Title/Abstract])) OR (Extracorporeal Life Support[Title/Abstract])) OR (Extracorporeal Life Supports[Title/Abstract])) OR (Life Support, Extracorporeal[Title/Abstract])) OR (Venoarterial ECMO[Title/Abstract])) OR (ECMO, Venoarterial[Title/Abstract])) OR (Venoarterial ECMOs[Title/Abstract])) OR (Venoarterial Extracorporeal Membrane Oxygenation[Title/Abstract])) OR (Venovenous ECMO[Title/Abstract])) OR (ECMO, Venovenous[Title/Abstract])) OR (Venovenous ECMOs[Title/Abstract])) OR (Venovenous Extracorporeal Membrane Oxygenation[Title/Abstract])**

**#3 Intra-Aortic Balloon Pumping[MeSH Terms]**

**#4** **((((((Intra Aortic Balloon Pumping[Title/Abstract]) OR (Intraaortic Balloon Pumping[Title/Abstract])) OR (Balloon Pumping, Intraaortic[Title/Abstract])) OR (Pumping, Intraaortic Balloon[Title/Abstract])) OR (Pumping, Intra-Aortic Balloon[Title/Abstract])) OR (Balloon Pumping, Intra-Aortic[Title/Abstract])) OR (Pumping, Intra Aortic Balloon[Title/Abstract])**

#5Cardiogenic Shock **[MeSH Terms]**

**#6 ((((((Arrest, Heart[Title/Abstract]) OR (Cardiac Arrest[Title/Abstract])) OR (Arrest, Cardiac[Title/Abstract])) OR (Asystole[Title/Abstract])) OR (Asystoles[Title/Abstract])) OR (Cardiopulmonary Arrest[Title/Abstract])) OR (Arrest, Cardiopulmonary[Title/Abstract])**

**#7 (#1 OR #2) AND (#3 OR #4) AND (#5OR #6)**


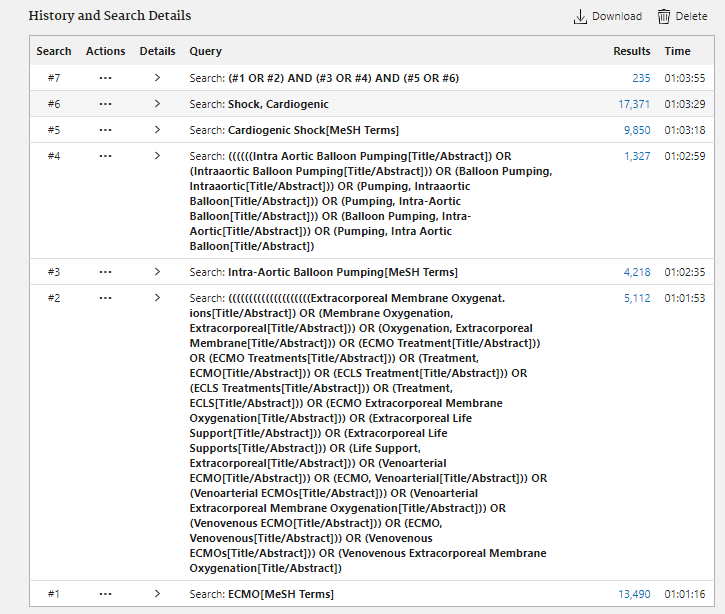


**Web of science (n=506)**


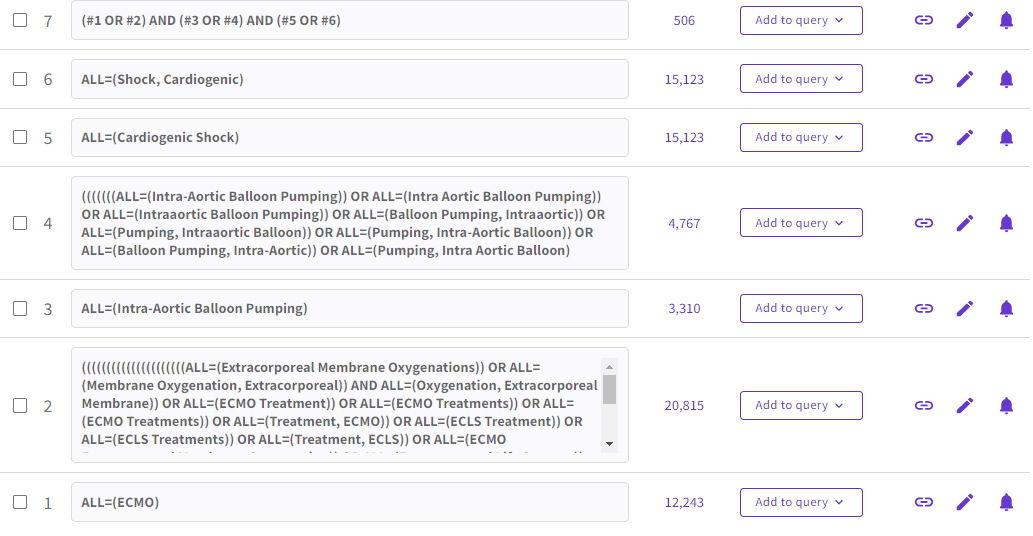


**Cochrane(n=0)**


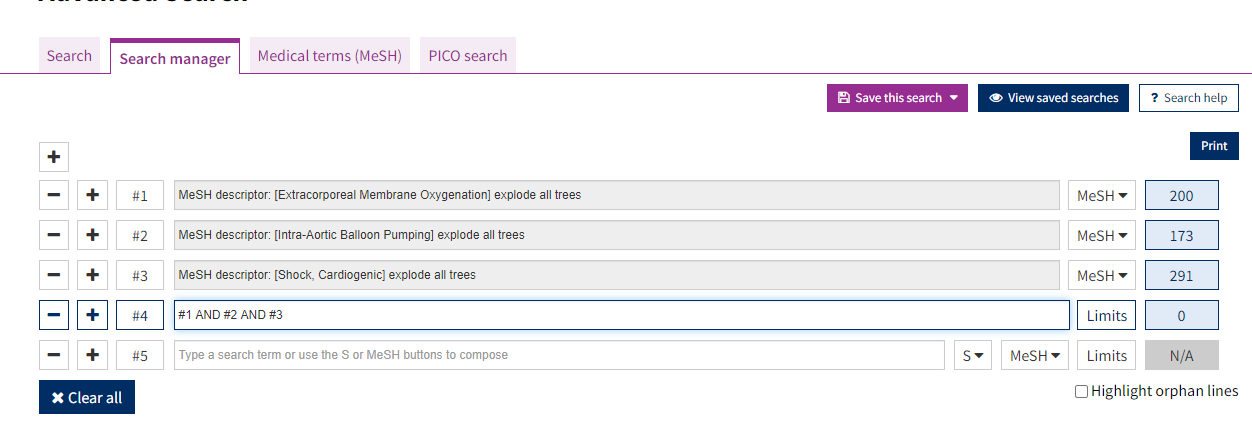


**Embase(n=57)**


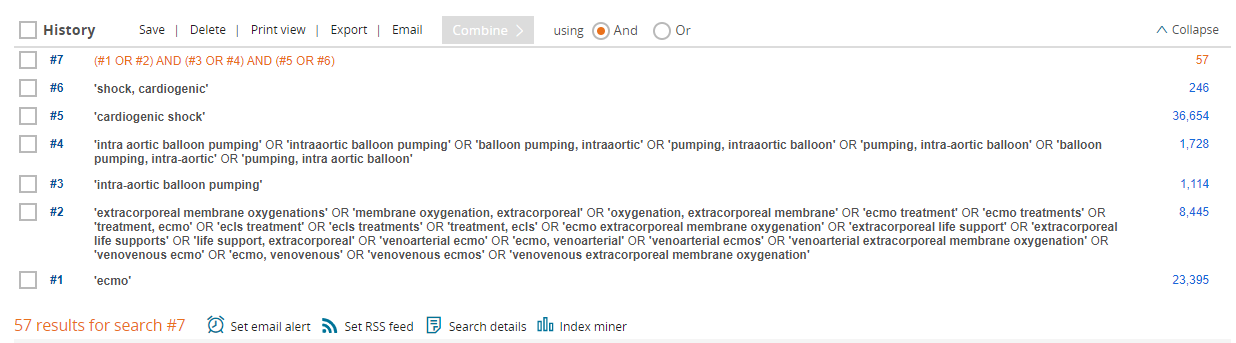


Reviewer 2

7.Thank you for your suggestion. We have made the assessment of quality by Gradepro, and the result is as follows.


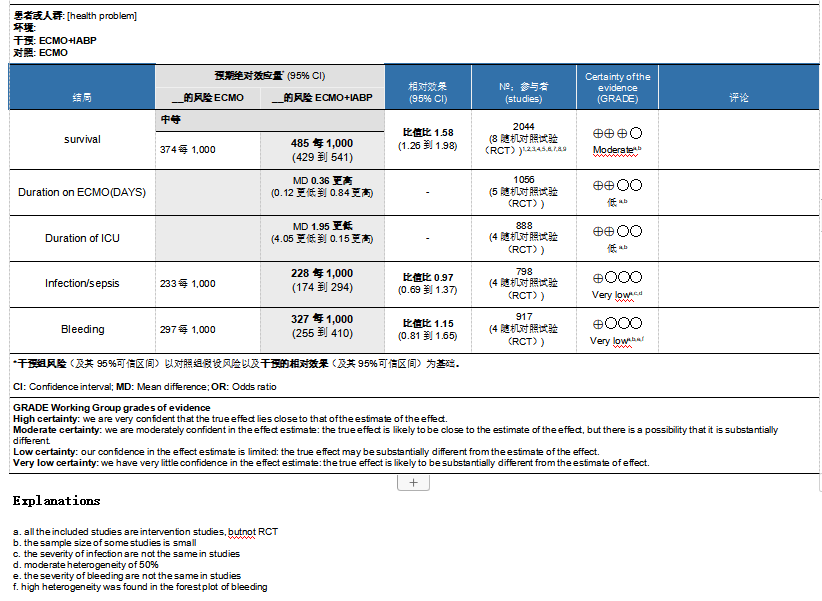

Supplement: Supplementary file 1 [file Table_1.DOCX]
